# The relationship between women’s breastfeeding empowerment and conformity to feminine norms

**DOI:** 10.1186/s12884-023-05628-z

**Published:** 2023-04-25

**Authors:** Maryam Dehghani, Ashraf Kazemi, Zeinab Heidari, Fatemeh Mohammadi

**Affiliations:** 1 Student Research Committee, School of Nursing and Midwifery, Isfahan University of Medical Sciences, Isfahan, Iran; 2 Nursing and Midwifery Care Research Center, School of Nursing and Midwifery, Isfahan University of Medical Sciences, Isfahan, Iran; 3 Reproductive Sciences and Sexual Health Research Center, Isfahan University of Medical Sciences, Isfahan, Iran; 4 Reproductive Sciences and Sexual Health Research Center, Isfahan University of Medical Sciences, Hezar Jerib Street, Isfahan, 8174673461 Iran

**Keywords:** Breastfeeding, Empowerment, Conformity, Feminine, Norms, Women

## Abstract

**Background:**

Women empowerment is effective in successful breastfeeding. Hence,identifying the relationship between psychosocial factors, such as acceptance of feminine norms, and empowerment can be beneficial in designing interventions.. Therefore, this study aimed to determine the relationship between breastfeeding empowerment and conformity to feminine norms.

**Methods:**

This cross-sectional study was conducted on 288 primiparous mothers in the postpartum period using validated questionnaires of conformity to gender norms and breastfeeding empowerment in the following domains: “sufficient knowledge and skills for breastfeeding,” “a sense of breastfeeding competence,” “conscious belief in the value of breastfeeding,” “overcoming breastfeeding problems,” “negotiation and obtaining family support” and “self-efficacy in breastfeeding” which were completed through the self-report method. Data were analyzed using the multivariate linear regression test.

**Results:**

The mean score of ‘conformity to feminine norms’ and ‘breastfeeding empowerment’ were 142.39 and 144.14, respectively. The score of breastfeeding empowerment was positively related to conformity to feminine norms (*p* = 0.003). Among the dimensions of breastfeeding empowerment, ‘mothers’ adequate knowledge and skills for breastfeeding’ (*p* = 0.001), ‘belief in the value of breastfeeding’ (*p* = 0.008), and ‘negotiation and obtaining family support’ (*p* = 0.01) were positively related to conformity to feminine norms.

**Conclusions:**

The results indicate a positive relationship between the level of conformity to feminine norms and breastfeeding empowerment. Accordingly, it is recommended that supporting breastfeeding as a valuable role of women be considered in programs designed to improve breastfeeding empowerment.

## Background

Breastfeeding is the optimal method of feeding a baby, which has countless benefits for the infant’s growth, development, and health maintenance. Feeding a child with breast milk reduces the possibility of contracting many diseases, including asthma, obesity, type 1 diabetes, severe lower respiratory disease, acute otitis media, sudden infant death syndrome (SIDS), dental malocclusion, and gastrointestinal infections [1]. Breastfeeding is also effective in preventing maternal diseases. Longer durations of breastfeeding contribute to the health and well-being of mothers; it reduces the risk of ovarian and breast cancer and helps to space between pregnancies [2].

Breastfeeding is of particular importance in developing countries, and planning to promote exclusive breastfeeding and its continuation until the end of one year of age requires identifying the associated factors [3]. Various factors, including breastfeeding experiences, gestational age at delivery time, type of delivery, the infant’s health status, and women’s breastfeeding empowerment, affect the initiation and duration of breastfeeding [4, 5].

Among the identified factors affecting breastfeeding, the mother’s empowerment is an essential indicator in predicting successful exclusive breastfeeding [6]. Kang and etal.'s study showed that in women who were empowered to breastfeed, the rate of exclusive breastfeeding of 4, 8, and 12-month old babies was higher than in the control group, and empowered women were more capable of solving breastfeeding problems than the other group [7]. The concept of empowerment in breastfeeding conforms to the general meaning of enabling, which means the process of improving decision-making ability and efficacy leading to a better position, which can be formed under the influence of individual factors and issues caused by an individual’s interaction with the environment [8].

From one point of view, breastfeeding is part of motherhood and being a mother is part of a woman's identity as an adult [9]. Today, concepts related to motherhood have changed, and women who behave according to new concepts and values constitute a greater proportion [10]. Gerson stated that today a mother is known as an ideal mother who, in addition to fulfilling household and motherly duties, plays a role in career fields for the growth and excellence of her personal and social talents [11]. Gillespie has reported that in recent years, women do not want to follow the dominant definitions of femininity, and they have redefined the female identity, which includes different norms compared to the previous identity [12]. This identity change is associated with differences in playing gender roles even affected women's views on the value of biological gender roles, such as breastfeeding.

Feminine roles are formed in the context of social norms. For each social role, norms define specific behavioral approaches [13]. Playing social roles requires accepting the norms related to them, and the behavior that fulfills them is called conformity. Studies have shown that conformity to gender norms influences health-related behaviors, including alcohol and tobacco consumption, sleeping pattern, and chronic diseases [14–16].

Various studies have investigated the extent of women's compliance with gender norms in different societies [17, 18]. The results showed that Swedish women had less conformity with feminine norms than their foreign counterparts [19]. Similarly, the studies conducted in Iran indicate changes in gender-related norms. Safiri and Maaroufpour (2018) consider the conflict in playing the gender role and lack of sexual creating collective identity conflict in women [20]. Farahmand and Tavangar stated that women’s identity in the social framework of one of the traditional and religious cities of Iran (Yazd) was likewise transitioning from traditional to modern, and most women in this city had an intermediate identity [21]. According to a study by Kanani and et al., in another city in Iran (Rasht), there was a difference in gender identity between two generations, namely girls and mothers. Because the girls used social media, their gender identity has changed [22].

Based on the evidence, it is expected that by changing the social concepts of gender roles and accepting the existing ones, women's ability to play roles related to female biology, as a part of the gender role, will also face challenges. Moreover, conformity to gender norms is a cultural-dependent variable that needs to be assessed in different societies. Therefore, the present study was conducted to determine the relationship between breastfeeding empowerment and conformity to feminine norms in primiparous mothers receiving postpartum care.

## Methods

This was a cross-sectional study whose protocol was approved by the Medical Research Ethics Committee of Isfahan University of Medical Sciences. The research population included primiparous women who had given birth two weeks. The inclusion criteria included Iranian nationality, reading and writing ability, no psychological disorders, intended pregnancy, giving birth at a gestational age of 37 weeks or more, no pregnancy with assisted reproductive methods, a healthy newborn, and no temporary or permanent contraindications to breastfeeding. The study setting was Isfahan, one of the metropolises of Iran. According to the latest census (2016), the population of this city is about 2 million and 20 thousand people, and the total fertility rate in this city is 1.49 [23]. In this city, all pregnant women and those who have given birth are covered by the care services of comprehensive health care centers. These centers provide mothers with all the training, counseling, and support services related to breastfeeding.

### Sample size calculation

Considering the 95% confidence interval and 80% power, and estimated standard deviation of breastfeeding empowerment equal to 13 [6], the sample size was obtained as 288 individuals.

### Data collection

Data were collected using demographic information, breastfeeding empowerment, and conformity to feminine norms questionnaires. The demographic information checklist included age, Level of education, employment type, family income level, and length of time that passed since the marriage. The type of delivery and sex of the child were also recorded. Empowerment in breastfeeding was evaluated using Heydari et al.’s breastfeeding empowerment questionnaire [24]. The questionnaire consists of 37 items evaluating empowerment in seven domains, including “sufficient knowledge and skills for breastfeeding (11 items),” “feeling of breastfeeding adequacy (4 items),” “conscious belief in the value of breastfeeding (7 items),” “overcoming breastfeeding problems (7 items),” “negotiation and gaining family support (5 items),” and “self-efficacy in breastfeeding (4 items).” The items have been compiled on a 5-point Likert scale (from 1 = completely disagree to 5 = completely agree), and the total score range of the questionnaire is 37–185. Obtaining a higher score from this questionnaire signifies a mother’s higher breastfeeding empowerment. In the investigations, the internal consistency of the questionnaire was reported to be higher than 0.70 using Cronbach’s alpha 0.87 and the validity index of all items was above 0.87 [24].

Conformity to feminine norms was evaluated using Pernet and Moradi’s questionnaire, extracted from the initial questionnaire of conformity to feminine norms developed by Mahalik [17]. This questionnaire contains questions on different dimensions of feminine norms, including modesty, attention to appearance, thinness, sexual fidelity, romantic relationships, relationship with children, friendships and, household affairs for assessment of attitudes, beliefs and behaviors related to traditional and non-traditional feminine gender roles. Pernet and Moradi’s questionnaire was translated and retranslated to be used in this study, and its face validity was examined qualitatively by ten faculty members of the Departments of Midwifery and Psychology. Afterward, its content validity was assessed and confirmed based on 15 experts’ opinions and by calculating CVI and CVR (the remaining items in the questionnaire had CVI above 0.81 and CVR above 0.60). Moreover, the reliability of this instrument was measured and confirmed by the test–retest method (*r* = 0.88) and internal consistency with Cronbach’s alpha (α = 0.74). This questionnaire has 50 items rated on a 5-point Likert scale (1 = completely disagree to 5 = completely agree), and scores range was between 50 and 200. A higher score indicates greater conformity to feminine norms.

The study was conducted with the participation of 288 primiparous women receiving postpartum care at the end of the second week of postpartum, in 2022 Feb-Aug. A stratified random sampling method was employed. Four geographical regions of Isfahan were considered strata, and eight healthcare centers (2 centers from each region) were randomly selected. In each center, sampling was performed using the convenience method. In this way, the samples were selected from the women who came to receive postpartum care services during the researcher’s presence at the center. After checking the inclusion criteria in the mothers, the researcher explained the study objectives and conducting method to mothers and invited them to participate. All participants signed an informed consent form before participating in the study. They were assured of the confidentiality of their information, and all questionnaires were completed anonymously. Finally, three questionnaires of demographic information, breastfeeding empowerment, and conformity to feminine norms were provided to them to be completed.

### Statistical analyses

One hundred percent of the questionnaires (without missing data) were included in the analysis. Data were analyzed using SPSS software version 19, descriptive statistics and statistical tests including independent t-test, Pearson’s correlation coefficient, and multivariate linear regression by modifying the results for underlying variables (level of education, employment status, family income status, delivery type, and baby’s sex). After performing the correlation and t-test (based on the variable type), the variables with a significant relationship with conformity to feminine norms, including mother’s age (*r* = 0.25), educational level (*r* = -0.17), family income (*r* = -0.18), and employment status (t = 4.67, *p* < 0.001), were recognized as confounding and entered into the regression model. Finally, the relationship between the main variables (breastfeeding empowerment and conformity to feminine norms) was calculated by adjusting the results for confounding variables and performing regression. The significance level for the tests was considered less than 0.05.

## Results

Statistical analysis was performed on the data of 288 mothers with a mean age (SD) of 26.03(5.42) years, ranging from 15 to 40. The results showed that the average time (SD) elapsed since marriage was 3.45(2.80) years, ranging from 1 to 18. Most mothers had university education and were housewife with adequate income to make ends meet. The type of delivery in more than half of the mothers was natural, and most babies were female (Table 1). The mean score of conformity to feminine norms and breastfeeding empowerment were 142.39 and 144.14, respectively. The average score of breastfeeding empowerment dimensions are also mentioned in Table 2.

**Table 1:** Frequency distribution of study participants based on demographic and fertility characteristics

Variable	Class	Number	Percent
**Level of Education**	Elementary	13	4.6
	Guidance	52	18.2
	High school	83	29.1
	University	137	48.1
**Employment status**	Housewife	210	73.7
	Employed	75	26.3
**Family income status**	Within the limits of expenses	232	81.4
	Less than outside	18	6.3
	More than expenses	35	12.5
**Type of delivery**	NVD	168	58.9
	C/S	117	41.1
**Baby sex**	Girl	159	55.8
	Boy	126	44.2

*Abbreviations*: *NVD* Natural vaginal delivery, *C/S* Cesarean section

**Table 2:** Descriptive indicators of Conformity to feminine norms and breastfeeding empowerment and its dimensions

Variable		Mean	SD	Possible score range
**Conformity to feminine norms**		142.39	10.99	50–200
**Breastfeeding Empowerment**		144.14	19.61	37–185
**Domains**	**Sufficient knowledge and skills for breastfeeding**	43.72	6.34	11-55
	**A sense of breastfeeding competence**	14.17	3.45	4-25
	**Conscious belief in the value of breastfeeding**	28.69	4.19	7-35
	**Overcoming breastfeeding problems**	25.84	5.3	7-35
	**Negotiation and obtaining family support**	19.84	3.64	5-25
	**Self-efficacy in breastfeeding**	11.89	2.5	3-15

The correlation test and regression model showed that mothers’ sufficient knowledge and skills for breastfeeding (*p* = 0.001, *r* = 0.23, CI = 0.06–0.23), belief in the value of breastfeeding (*p* = 0.03, *r* = 0.15, CI = 0.02–0.13), negotiation and gaining family support (*p* = 0.01, *r* = 0.17, CI = 0.01–0.10), and the total score of breastfeeding empowerment (*p* = 0.01, *r* = 0.17, CI = 0.14–0.65) had a direct and significant relationship with conformity to feminine norms. However, there was no relationship between conformity to feminine norms and other domains of breastfeeding empowerment (*p* > 0.05) (Table 3).

**Table 3:** Determining the relationship between conformity to feminine norms and breastfeeding empowerment and its domains in the regression model by adjusting the results for the mother’s age, natural delivery, female infant, sufficient income, and being a housewife

	Total score of breastfeeding empowerment			Self-efficacy in breastfeeding			Negotiation and obtaining family support			Overcoming breastfeeding problems			Conscious belief in the value of breastfeeding			A sense of breastfeeding competence			Sufficient knowledge and skills for breastfeeding		
	R^2^_Adj_ = 0.05_,_ *p* = 0.01, F = 2.74			R^2^_Adj_ = 0.01, *p*= 0.19,F = 1.45			R^2^_Adj_ = 0.06, *p*= 0.006,F = 3.14			R^2^_Adj_ = 0.001, *p*= 0.40,F = 1.03			R^2^_Adj_ = 0.04, *p*= 0.02, F = 2.54			R^2^_Adj_ = 0.04, *p* = 0.03, F = 2.29			R^2^_Adj_ = 0.04, *p*= 0.01, F = 2.60		
	Beta	CI 95%		Beta	CI 95%		Beta	CI 95%		Beta	CI 95%		Beta	CI 95%		Beta	CI 95%		Beta	CI 95%	
**Age**	0.03	-0.46	0.69	0.05	-0.05	0.10	-0.05	-0.13	0.06	0.07	-0.09	0.24	0.02	-0.10	0.14	0.02	-0.09	0.12	0.13	-0.03	0.33
**Natural delivery**	-0.02	-6.71	4.74	-0.01	-0.87	0.68	-0.01	-1.08	0.94	0.02	-1.40	1.90	-0.09	-1.96	0.48	-0.09	-1.76	0.40	0.10	-0.53	3.12
**female infant**	-0.06	-7.69	2.85	-0.06	-1.04	0.39	-0.11	-1.67	0.18	0.02	-1.24	1.81	-0.65	-1.63	0.61	0.008	-1.05	0.93	-0.05	-2.30	1.05
**sufficient income**	-0.15*	-27.07	0.04	-0.15*	-3.80	-0.06	-0.18**	-5.56	-0.70	-0.13	-7.82	0.23	-0.06	-4.21	1.66	0.05	-3.50	1.70	-0.02	-5.17	3.58
**being a housewife**	0.09	-2.92	9.93	0.09	-0.44	1.38	0.17*	-0.01	2.33	0.08	-0.92	2.96	0.17*	-0.02	2.81	0.19*	0.18	2.70	0.05	-1.45	2.77
**Conformity to feminine norms**	0.23*	0.14	0.65	0.11	-0.01	0.06	0.19**	0.01	0.10	0.06	-0.04	0.10	0.20**	0.02	0.13	0.17	0	0.10	0.27**	0.06	0.23

* *p*< 0.05

**p = 0.01

Investigating the relationship between conformity to feminine norms and individual characteristics, an inverse and significant relationship was found between mothers ‘age and conformity to feminine norms (*p* = 0.001, *r* = -0.25, CI = -0/34- -0/11). As the age increased, mothers’ conformity to feminine norms decreased. Mothers’ educational level had an inverse relationship with conformity to feminine norms (*p* = 0.16, *r* = -0.17, CI = -0.29- -0.07); however, the relationship was not statistically significant. Moreover, this test showed that the family’s income status had an inverse and significant relationship with conformity to feminine norms, i.e. (*p* = 0.01, *r* = -0/18, CI + -0.32- -0/07), with the increase in the income level, the mean score of conformity to feminine norms decreased.

According to the independent t-test results, the mean score of conformity to feminine norms in employed mothers and housewives was statistically different (*p* < 0.001, t = 4.67, CI = 3.95–9.56); the mean score of conformity to feminine norms in housewives (144.17) was significantly higher than that of employed mothers (137.41).

## Discussion

The present study aimed to determine the relationship between breastfeeding empowerment and conformity to feminine norms. The results showed that the total score of breastfeeding empowerment and some of its domains (knowledge and skill of breastfeeding, belief in the value of breastfeeding, and negotiation and obtaining family support) had a direct and significant relationship with conformity to feminine norms. None of the individual characteristics were related to breastfeeding empowerment.

As the data analysis indicated, the score of knowledge and skill of breastfeeding, belief in the value of breastfeeding, and breastfeeding empowerment increased as the score of conformity to feminine norms increased. Women with higher conformity to feminine norms were better adapted to issues related to this role [25]. Moreover, socially-defined feminine norms persuaded women to perform or refrain from certain behaviors. Some of these norms were an obstacle to breastfeeding. Davis et al. have stated that in America, prudency is considered a social norm for African-American women, and it conflicts with breastfeeding, due to which the breasts might be visible. Conformity to this norm reduces women’s empowerment to opt for breastfeeding [26].

On the other hand, when the norms impose breastfeeding on women, non-conformity to the norms causes society’s unsupportive behavior and a sense of shame in the mother. In such a situation, some women breastfeed the child against their will, which might lead to low quality or discontinuity of breastfeeding [27]. Therefore, studies suggest that it is essential to influence women’s preferences and value systems to empower them in breastfeeding [28]. If women are empowered to breastfeed, they capable to be the defender of breastfeeding, which provides them with a new framework for interpreting their abhorrence of breastfeeding or others’ reactions [29]. Moreover, they become more powerful in overcoming the problems and hardships of breastfeeding [30].

Based on the results, no individual characteristics (mothers’ age, education level, occupation, and family income status) had a relationship with breastfeeding empowerment. Similarly, in Moafi et al.’s study, the score of self-efficacy in breastfeeding was not significantly different in relation to any of the demographic characteristics [8, 31, 32]. However, Hadisuyatmana et al. have reported higher education and socioeconomic status as facilitating factors of breastfeeding empowerment [33]. Davis et al. likewise stated that mothers with higher education and better financial status felt more empowered to select breastfeeding and react to the health personnel’s behavior when receiving services [26]. Since the study by Moafi et al. was carried out in Iran and had similar results to the present study, it might be stated that other factors, such as mothers’ attitudes toward breastfeeding, perceived family support, education on breastfeeding, and the capability to manage breastfeeding complications, influence women’s empowerment in the sociocultural context of the mentioned country [6].

However, various studies have shown that both empowerment and self-efficacy are directly related to compliance with health behaviors (like breastfeeding) [6, 34] and the use of health services [35, 36]. It should be noted that self-efficacy and empowerment are not synonymous. It is important to pay attention to the concepts in order to clarify their differences. Empowerment means independence in decision-making, having the right to choose and access opportunities [37], but self-efficacy is a factor for valuing personal abilities and having the determination to change health behavior by the individual and persist on it [38]. So women are empowered when they have independence in making decisions regarding breastfeeding type and have access to various options to select from. After they decide to breastfeed, the ability to overcome the obstacles of breastfeeding and the determination to continue is self-efficacy.

Regarding the mothers in the present study, the score of conformity to feminine norms decreased as age and family income increased. Concerning family income, studies have shown that conformity to feminine norms is more desirable in societies with lower socioeconomic status. The study by Marvan et al. (in Mexico) showed that girls were persuaded to play traditional female social roles from childhood in a society with lower socioeconomic status. The social norms defined for these roles include having menstrual cycle, pregnancy, childbirth, breastfeeding and childbearing, which has caused these women to have fewer negative experiences in dealing with life events than their peers [39].

Regarding the relationship between age and conformity to feminine norms, our study showed that conformity to feminine norms decreased with with increasing age. However, the study conducted by Simonen in Finland showed that older women were more subject to traditional norms of gender roles (including moderation, decency, respect, and more self-control). In contrast, young women had newer feminine norms (including independence, gender equality, and decision-making to perform affairs based on personal desire). Young women behave according to these new norms [40]. Another study has similarly shown that with increasing age, conforming to existing norms changes, and in some cases, younger women redefine the concepts of existing norms. For instance, in the younger generations of Swedish women, femininity is defined more by physical characteristics, such as physical fitness and facial beauty, than psychological factors [19]. Based on the mentioned studies, to discover the reason for the decrease in conformity to feminine norms at an older age, conducting more research is necessary in order to explain the feminine norms in different generations of Iranian women.

In the present study, housewive’ mean score of conformity to feminine norms was significantly higher than that of working mothers. Farahmand and Tawanger have reported that identity dynamics (transition from traditional to modern female identity) are higher in working women than in unemployed women. When the score of female identity dynamics increases, the existing norms lose their function, making it more challenging to conform to [21]. A study by Salway et al. likewise indicates that employment challenges women’s traditional identity, which is in line with the results of the present study [41].

The strength of the present study is the use of specific questionnaires on breastfeeding empowerment and conformity to feminine norms to measure the research variables. The limitation of the present study was the small number of samples. Besides, the investigated variables are dependent on the sociocultural background, which reduces the generalizability of the results. Consequently, conducting more studies with larger sample sizes and investigating the variables in different societies seems necessary.

## Conclusion

The present study shows that breastfeeding empowerment is directly correlated with conformity to feminine norms, and breastfeeding is a behavior rooted in gender norms. Therefore, this factor needs to be taken into account when designing interventions and programs to promote breastfeeding. It is suggested to design and implement comprehensive long-term programs to strengthen the social valuation of breastfeeding and make it pleasurable as a part of feminine roles. Consequently, as future mothers, today’s girls are prepared to accept breastfeeding.

## Data Availability

The datasets generated and/or analyzed during the current study are available from the corresponding author (FM) on reasonable request.

## Abbreviation

SIDS: Sudden infant death syndrome
NVD: Natural vaginal delivery
C/S: Cesarean section
CI: Confidence interval
